# Rheumatoid factor production is genetically and molecularly distinct from rheumatoid arthritis

**DOI:** 10.1172/JCI208390

**Published:** 2026-06-30

**Authors:** Mehmet Hocaoğlu, Amr H. Sawalha

**Affiliations:** 1 Division of Rheumatology and Clinical Immunology, Department of Medicine, University of Pittsburgh, Pittsburgh, Pennsylvania, USA.; 2 Lupus Center of Excellence, University of Pittsburgh School of Medicine, Pittsburgh, Pennsylvania, USA.; 3 Division of Rheumatology, Department of Pediatrics, and; 4 Department of Immunology, University of Pittsburgh, Pittsburgh, Pennsylvania, USA.

**Keywords:** Autoimmunity, Genetics, Rheumatology

## Abstract

**BACKGROUND:**

Rheumatoid factor (RF) autoantibodies are highly prevalent, yet the molecular determinants of RF development and its progression to rheumatoid arthritis (RA) remain poorly understood. Here, we define the genetic, phenotypic, and molecular architecture of RF and its progression to RA.

**METHODS:**

A total of 469,036 UK Biobank participants with RF testing and 76 Allen Institute for Immunology–University of California, San Diego–University of Colorado Transition to Rheumatoid Arthritis (ALTRA) cohort individuals were studied. Phenome-wide association studies (PheWAS), GWAS, and proteome-wide association studies (PWAS) compared RF^+^ individuals who did not have autoimmune disease with RF^–^ control individuals. Single-cell RNA-seq enabled pseudobulk differential expression and cytokine signature enrichment analyses.

**RESULTS:**

RF seroprevalence was 9.3% and longitudinally stable in 94.5% of individuals. PheWAS identified 48 significant associations, led by chronic viral hepatitis (OR 4.8), hypersensitivity pneumonitis (OR 3.6), bronchiectasis (OR 1.9), and COPD (OR 1.4). GWAS of 24,216 RF^+^ individuals revealed 29 independent loci; the strongest signal was in the extended HLA region (OR 1.45, *P* = 5.4 × 10^–221^). Non-HLA loci converged on B cell homeostasis genes (*ETS1*, *BACH2*, *PAX5*, *TNFRSF13B*, *FCGR2A*). RF^+^ individuals did not carry elevated RA polygenic risk. Proteomics profiling identified 153 differentially abundant proteins enriched for humoral immunity and IFN-induced chemokines, with 79% showing dose response relationships across titers. Progression to RA involved a shift toward activating tissue-damaging inflammatory pathways rather than amplification of the RF signature. Single-cell transcriptomics of RF^+^ individuals without RA localized dysregulation to memory B cells, with downregulation of inhibitory genes (*FCGR2B*, *BACH2*, *FOXP1*) and upregulation of activation markers.

**CONCLUSION:**

RF production is governed by HLA class II and B cell regulatory loci, associated with mucosal inflammation, and genetically and molecularly distinct from RA.

**FUNDING:**

This study was supported by the Rheumatoid Arthritis Research Program Grant from the Arthritis Foundation.

## Abstract 2

Rheumatoid factor has unique genetic and immune features, separate from rheumatoid arthritis, helping explain why most carriers remain healthy

## Introduction

Rheumatoid factor (RF), an antibody against the Fc portion of IgG, is one of the most common autoantibodies in the general population (1, 2). RF has a strong association with rheumatoid arthritis (RA) and may precede the disease onset by several years (3). However, RF shows limited specificity for RA and can be observed in other autoimmune and infectious diseases, such as viral hepatitis (4–6). Furthermore, most individuals with RF do not develop RA or other autoimmune diseases, and RF positivity may be transient during infections, possibly reflecting physiological roles in clearing immune complexes (7).

Emerging data show that individuals with RA-related autoantibodies, such as anti–cyclic citrullinated peptide antibodies (ACPAs), harbor substantial immune dysregulation prior to the clinical onset of RA (8). ACPA is highly specific for RA and is much less common in the general population compared with RF (1, 9, 10). Whether individuals with RF positivity, especially those who are otherwise asymptomatic for autoimmune disease, also harbor substantial immune dysregulation remains incompletely understood. Moreover, while the genetic risk factors of RA are well studied (11), there is limited understanding of the genetic basis of RF production and how it relates to RA.

In this study, we comprehensively characterized the molecular signature of RF by performing GWAS, phenome-wide association studies (PheWAS), proteome-wide association studies (PWAS), and transcriptome-wide association studies (TWAS) (Figure 1). We uncovered the genetic basis of RF production and show that RF exhibited an architecture genetically and molecularly distinct from that of RA, marked by selective immune activation that occurred without tissue-destructive inflammatory responses.

## Results

### RF production is predominantly low titer and prevalent in the general population.

The seroprevalence of RF positivity was 9.3% among 469,036 participants with baseline RF testing in the UK Biobank. RF production was predominantly low titer: 6.8% of UK Biobank participants had titers below 3 times (3×) the upper limit of normal (ULN), whereas higher titers were progressively less common (2.5% >3× ULN; 1.1% >6× ULN; 0.5% >12× ULN). There were only negligible differences across RF titer subgroups in demographic characteristics (Table 1). We observed markedly increased RA prevalence with rising RF titers, while the prevalence of overall clinical autoimmunity excluding RA was comparable (Table 1).

### RF serostatus shows longitudinal stability with seroconversions concentrating at low titers.

We next investigated whether RF positivity is transient or stable over time. Repeat RF testing was available for 16,660 individuals (mean interval 4.3 ± 0.92 years). Overall serostatus was stable in 94.5% of the repeat tests (*n* = 15,736). Twenty-six percent of baseline seropositive individuals (*n* = 387) converted to seronegative status, and 3.5% of baseline seronegative individuals (*n* = 537) converted to seropositive status. Seroconversions were concentrated in the lowest titer range: 93% of the seropositive converters transitioned to the less-than 3× ULN category, whereas 97% of seronegative converters had baseline titers below 3× ULN (Supplemental Figure 1; supplemental material available online with this article; https://doi.org/10.1172/JCI208390DS1).

### RF is associated with conditions characterized by chronic infections and mucosal inflammation.

To identify conditions associated with RF production beyond clinical autoimmunity, we performed a PheWAS after excluding individuals with autoimmune diseases. For this analysis, there were 36,049 RF^+^ individuals and 383,422 RF^–^ individuals with available phenotypic data. We identified 48 phenotypes with a significant RF association at an FDR of 0.1 (Figure 2). The most significant association was chronic viral hepatitis (OR 4.8, adjusted *P* = 3.4 × 10^–23^) with several associated viral hepatitis-related signals (Supplemental Table 1). Beyond hepatic signals, the most prominent cluster of associations involved several respiratory disease phenotypes. Hypersensitivity pneumonitis (OR 3.6, adjusted *P* = 6.6 × 10^–4^) had the largest effect size across these disorders followed by bronchiectasis (OR 1.9, adjusted *P* = 2 × 10^–12^), interstitial lung disease (OR 1.7, adjusted *P* = 5.8 × 10^–3^), and COPD (OR 1.4, adjusted *P* = 5.1 × 10^–14^). Other prominent signals included sepsis, anogenital infections, pregnancy complications, and lung neoplasms (Supplemental Table 1).

### Genetic architecture of asymptomatic RF production implicates the HLA region and B cell regulation.

After quality controls, we analyzed 7,225,682 genetic variants in 24,216 asymptomatic RF^+^ individuals and 244,786 RF^–^ controls for a GWAS. There were 17,188 genetic variants across 29 genetic loci associated with asymptomatic RF production at the genome-wide significance level (Figure 3). A total of 16,058 of these variants were localized within the extended HLA region (chromosome [chr] 6, 25–34 Mb) (Supplemental Figure 2). The top hit in the HLA region was the SNP rs3129959 (OR 1.45, 95% CI 1.42–1.48, *P* = 5.4 × 10^–221^), while the classical allele with the strongest association was HLA-DQA1:05 (OR 1.3, 95% CI 1.28–1.33, *P* = 2.3 × 10^–135^). We observed statistically independent signals between rs3129959 and HLA-DQA1:05 in the pairwise conditional analysis (Supplemental Table 2). A protective missense association at *TNFRSF8* (rs2230624; OR 0.76, *P* = 2.6 × 10^–10^) locus showed the largest effect size among non-HLA variants (Supplemental Table 3). Other non-HLA risk loci mapped to genes responsible for B cell differentiation and identity (*ETS1*, *BACH2*, *PAX5*, *FOXP1*), the BAFF/TACI axis (*TNFRSF13B*, *TNFSF13B*), the Fc receptor and Ig biology (*FCGR2A*, *FCRL5*), immune cell development (*TBX21*, *ID2*, *GFI1*, *JAK1*), and costimulation (*TNFSF4*, *CD70*) (Supplemental Table 3). Furthermore, the RF-associated genetic loci showed a significant degree of protein-protein interactions (28 nodes/52 edges; expected number of edges, 6; *P* < 1 × 10^–16^, average node degree, 3.71) and were enriched for genetic pathways affecting the regulation of B cell activation (Supplemental Figure 3).

### Asymptomatic RF production is genetically distinct from RA.

We next assessed whether RF^+^ individuals without RA carried an increased RA genetic risk. Our analysis showed that individuals who were RF^+^ did not have a significant increase in RA genetic risk compared with RF^–^ controls, whereas RF^+^ RA cases expectedly had a markedly higher RA genetic risk (Figure 4A). Consistent with this observation, the RA polygenic risk score (PRS) demonstrated a good discrimination performance for identifying RF^+^ RA patients among RF^+^ individuals (ROC–AUC 0.76, 95% CI 0.75–0.77) (Figure 4B). We observed comparable findings in sensitivity analyses restricted to individuals with higher RF titers (>3× ULN and >12× ULN) (Supplemental Figure 4).

### RF production has a titer-dependent proteomics signature enriched for humoral immunity, antigen presentation, and IFN-induced chemokines.

After quality controls and exclusion of individuals with preexisting autoimmune diseases, there were 3,368 RF^+^ individuals and 34,155 RF^–^ individuals with available proteomics data in the UK Biobank. We identified 153 differentially abundant proteins between RF^+^ and RF^–^ individuals at a FDR of 1% (Figure 5). Across the serum proteome, we found that asymptomatic RF production was associated with an immune activation signature involving markers of antibody production and immune complex regulation (JCHAIN, MZB1, FCRL2, FCRL5, FCAMR, TRIM21), upregulation of IFN-induced chemokines (CXCL9, CXCL10, CXCL11, CXCL13, IFN-γ), and enhanced antigen presentation (HLA-E, CD74, CD80, CD27, TNFRSF9, LAG3, PDCD1) (Supplemental Table 4). In subgroup analyses, we identified 536 proteins with differential expression across RF titers, 79% of which showed monotonic relationships, with rising titers underscoring the titer-dependent nature of the RF proteomics signature (Supplemental Table 5). Notably, we observed a higher serum abundance of proteins that were associated with RF at the genetic level, such as TNFRSF8, TNFRSF13B, TNFSF13B, and FCRL5 (Supplemental Table 5). Furthermore, a sensitivity analysis excluding individuals with incident autoimmune disease development after cohort entry showed similar findings that further underscored the observation that RF production exhibits an independent proteomics signature pointing to a distinct immune dysregulation state (Supplemental Table 6).

### Progression to RA from RF production is marked by expansion of inflammatory and tissue-damaging pathways.

We next compared serum protein abundance in RF^+^ individuals without RA at the time of sample collection with individuals with RF^+^ RA and identified 747 differentially expressed proteins between the 2 groups (Supplemental Table 7). This showed that progression to RF^+^ RA was characterized by a broad expansion in both the number and magnitude of altered proteins, as asymptomatic RF production was characterized by comparatively limited proteomic perturbation with smaller effect sizes (*n* = 153) (Figure 5A). Furthermore, the RA-specific proteomics signature we identified had limited overlap with the proteomic alterations associated with RF production (*n* = 46). This suggests that proteomic dysregulation of RA accelerates at or near disease onset rather than increasing uniformly during the asymptomatic RF production phase. Characterizing the RA-specific proteomics signature (Figure 5B) revealed a dominant proteomics signature composed of key cytokines and synovial inflammation markers such as TNF (12), CCL7 (13), RRM2 (14), and MMP3 (15) that were largely unchanged with asymptomatic RF production but became strongly induced in RA, while other key cytokines such as IL-6, IL-10, and IL-12 (12, 16) increased progressively in abundance across both steps, suggesting early immune priming in RF that was further amplified in RA. Supporting these observations, enrichment analyses showed that pathways related to antigen presentation and adaptive immune system activation were upregulated in RF production, whereas inflammatory pathways such as neutrophil degranulation, extracellular matrix organization, and TNF-induced inflammation were more enriched in RF^+^ RA compared with RF production without RA (Supplemental Figure 5).

### RF production is associated with selective reprogramming of the memory B cell compartment.

To identify the immune cells and transcriptomics programs that are altered in asymptomatic RF production, we analyzed single-cell RNA-seq (scRNA-seq) data from the Allen Institute for Immunology–University of California, San Diego–University of Colorado Transition to Rheumatoid Arthritis (ALTRA) cohort. For this analysis, there were 36 RF^+^ and 40 RF^–^ individuals without preexisting RA with available single-cell sequencing data (Supplemental Table 8). Pseudobulk differential expression analyses showed that RF production was associated with limited transcriptional changes in contrast to the global dysregulation observed in ACPAs. There were 25 differentially expressed genes (DEGs) across 7 immune cell types in RF compared with 27,111 DEGs across 60 immune cell types in ACPAs at a FDR of 0.1 (Supplemental Figure 6). In subtype-specific analysis, we observed a greater degree of transcriptional dysregulation in IgA RF^+^ compared with IgM RF^+^ individuals. Furthermore, the majority of immune dysregulation observed in RF production was localized to the core memory B cell compartment. Specifically, in IgA RF, we found 180 DEGs across 7 cell subtypes, 165 of which were in core memory B cells compared with IgM RF, which had 36 DEGs across 9 cell subtypes, 13 of which were in core memory B cells (Supplemental Table 9). Core memory B cells, which represent the largest subpopulation of memory B cells characterized by markers such as *CD27* and *AIM2* (17), showed a coordinated transcriptional shift in B cell signaling with downregulation of inhibitory genes and upregulation of activation markers (Supplemental Table 9). Downregulated genes included canonical components of B cell receptor and inhibitory signaling program genes, including *BLNK*, *FCMR*, *FCGR2B*, *FCRL2*, and *CD72*. We also observed downregulation of multiple B cell regulatory genes, including *FOXP1*, *BACH2*, *BCL11A*, and *AFF3*. In contrast, we noted an upregulation of genes such as *CD86*, *PRKCD*, *TNIP2*, *SCIMP*, *ITGB1*, *ANXA2*, and *S100A10* linked to activation, costimulation, and cellular remodeling (Supplemental Table 9). Ig gene expression showed downregulation of *IGHM* and *IGHD*, with upregulation of *IGHA1*, which suggests a global shift toward IgA in the core memory B cell compartment (Supplemental Table 9). Further supporting the memory B cell reprogramming in RF production, differential abundance analysis of immune cell types showed increased levels of CD95^+^ memory B cells in the peripheral blood (β 0.55, adjusted *P* = 1.1 × 10^–2^), an effect observed for both IgA and IgM subtypes (Supplemental Table 10). Notably, we also observed a significant upregulation of *PPP1R14A* (log_2_FC = +1.08, *q* = 3 × 10^–5^) within the CD95^+^ memory B cell compartment (Supplemental Table 9). *PPP1R14A* inhibits protein phosphatase 1, and its upregulation could reflect a reprogramming of activation thresholds in CD95^+^ memory B cells. Our observation of the downregulation of *FOXP1* and *BACH2* genes in core memory B cells converged with the observation that germline variants in the same loci were associated with a protective effect on RF development (Supplemental Table 3). This suggests that the genetic variations in B cell activation thresholds could confer a propensity to develop RF antibodies.

### IgA RF and ACPA production exhibit shared and distinct transcriptional cytokine signatures.

To identify global cytokine programs associated with RF and ACPA status, we performed cytokine signature enrichment analysis across 23 immune cell types using the Human Cytokine Atlas. As the transcriptomic changes in RF were predominantly observed with the IgA subtype, we restricted this analysis to the IgA subtype of RF. We identified 18 qualifying cytokine signatures for ACPA and 16 for IgA RF, with 12 shared between both autoantibodies (Supplemental Table 11). IL-10 was the most broadly upregulated cytokine signature across both autoantibodies, with positive enrichment in 14 cell types for ACPA and in 13 cell types for IgA RF, converging with the serum proteomics observations (Figure 6). IL-4 and IL-21 were other cytokines concordantly upregulated in both autoantibody contexts. In contrast, the IFN suppression signature was evident in ACPA^+^ individuals, across IFN-β, IFN-ω, and IFN-γ. This IFN suppression was also partially present in IgA RF, in which IFN-γ and IFN-β were similarly downregulated but with heterogeneity across cell types, whereas IFN-α1 exhibited positive enrichment in DCs (Figure 6). Several T cell–associated cytokines, including IL-2 and IL-15, showed positive enrichment across multiple cell types in ACPA, whereas IgA RF displayed an inverted pattern with predominantly negative enrichment. IL-1β also displayed a discordance with positive enrichment in ACPA but predominantly negative enrichment in IgA RF (Figure 6). Collectively, these results reveal that both convergent and divergent cytokine-driven transcriptional programs underlie ACPA and IgA RF seropositivity.

## Discussion

In this study, we uncover the molecular determinants of asymptomatic RF production and its subsequent progression to clinical RA. We show that RF production and progression to RA involved distinct molecular pathways by integrating GWAS and PheWAS with serum proteomics, polygenic risk assessment, and single-cell transcriptomics of immune cells. Our findings indicate the potential of prioritizing the immune pathways for early prevention of autoimmunity by preventing RF development and subsequent RF-to-RA progression and enabling molecular risk stratification for RA progression across individuals with RF.

The classical allele with the strongest RF association in our study, HLA-DQA1:05, is a risk factor for the development of adalimumab antibodies (18, 19). The independent association of this classical allele with RF production potentially suggests a preferential binding of this HLA allele to Ig epitopes, which warrants further study. This HLA signal is different from the robust shared epitope association seen in RA (12). Prior literature showed that the genetic association with a shared epitope in RA is primarily seen in individuals with ACPAs (20). Differential HLA associations primarily driven by specific autoantibodies, regardless of clinical status, were also described previously (21). This suggests that divergent genetic signals in the HLA region might be the reason for antigen-specific susceptibility for RF, ACPA, and other specific autoantibody-related epitopes. Beyond the HLA region, individuals with asymptomatic RF positivity showed limited genetic overlap with RA. This suggests that the genetic risk for developing the asymptomatic phase of RA with the emergence of RF and subsequent progression to clinical RA might be conferred in an independent and additive fashion. As RF itself confers a marked future risk of RA (3), assessment of RA genetic risk among individuals who are RF^+^ might be a feasible strategy to stratify individuals for future RA risk.

The dynamic nature of the serum proteome between RF production and established RA suggests that there are potentially separate mechanisms associated with early and late stages of the RA pathology. Supporting this finding, prior research showed that patients with RA treated with immunosuppressive medications such as TNF inhibitors and methotrexate had comparable rates of autoreactive B cell clones before and after treatment, despite clinical improvement (22). Hence, RA protein biomarkers that we identify could potentially be grouped into early autoimmune markers associated with RF development and effector-phase markers associated with driving clinical symptoms. Accordingly, it is possible that early autoimmune markers could be used to predict future RA risk and be prioritized as drug candidates for early prevention efforts, while effector-phase markers might be higher-yield drug candidates for RA treatment after the disease onset. As illustrative examples, TNF and IL-6 are 2 significantly upregulated proteins in RA, when compared with asymptomatic RF production, that are targets for highly effective RA medications (23). On the other hand, drugs targeting proteins such as IFN-γ and TNFRSF13B, which are upregulated in RF production but do not significantly change in RA (as compared with RF), failed in previous clinical trials (24, 25).

A mucosal origin for RF and ACPA has long been suspected following several observations (26), such as the detection of RA-related autoantibodies in the sputum (27), an increased proportion of circulating IgA^+^ plasmablasts (28), and asymptomatic small airway inflammation observed on imaging in individuals with RA-related antibodies (29). In line with these observations, lung diseases such as COPD, bronchiectasis, and asthma were previously shown to have an association with RA (30, 31). Our results provide additional support to the mucosal origin hypothesis by demonstrating a strong association between RF production and these conditions independent of RA and other clinical autoimmunity. Furthermore, we expanded the link between mucosal inflammation and RF production by identifying hypersensitivity pneumonitis as one of the strongest associations with RF. A potential unifying mechanism for RF generation across these conditions could be the development of ectopic lymphoid tissues in the lung mucosa. Indeed, prior research demonstrated that inducible bronchus-associated lymphoid tissue (iBALT), an ectopic lymphoid lung tissue that develops in response to various insults, produces RF antibodies in the lungs of patients with RA (32). iBALT also develops in lung diseases such as hypersensitivity pneumonitis, COPD, and bronchiectasis, which might be the source of the RF production in these conditions (33–35). The association of RF with viral hepatitis, anogenital herpes infections, and pregnancy complications could also stem from inflammation in the biliary, genital, and anal mucosa within the framework of the mucosal origin hypothesis (36). Supporting this interpretation, an association between pregnancy complications and RF^+^ RA development was previously reported (37).

At the molecular level, the multiomics architecture of RF production converged primarily on the reprogramming of B cell activation thresholds. Notably, several RF risk loci we identified encode protein pairs with physical and/or functional interactions playing a role in B cell transcriptomic regulation: *PAX5*-*ETS1* (38), *ID2-GFI1* (39), *TNFRSF13B-TNFSF13B* (40), *PAX5-BACH2* (41), and *FOXP1*-*BACH2* (42). These factors collectively regulate the B cell activation thresholds and differentiation into antibody-secreting cells, suggesting that altered control of these pathways may be a key mechanism underlying RF generation (42–44). Consistent with this interpretation, immune complex receptors such as FCGR2B and Fc-like receptors that tune B cell activation thresholds were also implicated with RF at the genetic, proteomic, and transcriptomic levels (45, 46). This framework might also help explain the expansion of CD95^+^ memory B cells with RF that we observed in the ALTRA cohort. Although the developmental origin and function of CD95^+^ memory B cells remain incompletely defined, they have been proposed to represent an effector memory cell population enriched for recent germinal center emigrants (47). CD95 (Fas) has an essential role in the elimination of autoreactive B cells during germinal center selection (48). Hence, the genetic reprogramming of B cell activation thresholds that we observed might counteract the apoptotic effect of CD95 and favor the escape of autoreactive B cell clones. The spontaneous emergence of RF antibodies in lupus-prone MRL/*lpr* mice with Fas deficiency further supports a potential link between aberrant CD95 signaling and RF production (49).

The progressively greater transcriptomic dysregulation observed in IgM RF, IgA RF, and ACPA potentially suggests a stepwise model of disease evolution in at least a subset of patients. In this framework, autoreactive IgM RF^+^ B cell clones may first escape deletion because of genetic variations in B cell activation thresholds and stimulation through mucosal inflammation, followed by class switching to IgA RF and the subsequent emergence of ACPA before the onset of clinical disease. This framework is supported by the fact that IgA RF positivity in the absence of IgM RF is very rare (50), and IgA RF confers a greater RA risk than does IgM RF (51). Furthermore, the median duration of RF positivity before clinical onset was longer compared with ACPAs in a study of patients with RA who underwent blood sampling prior to disease onset (52). Nevertheless, another study showed discrepant findings, with ACPAs lasting longer prior to disease onset, which suggests the possibility that the broad immune activation seen in ACPAs might trigger RF production in certain contexts (53).

Interestingly, we observed an upregulation of IL-10 with RF, ACPAs, and established RA at both the proteomic and transcriptomic levels. Given its immunosuppressive effects, it is possible that IL-10 upregulation is a compensatory mechanism for the increased immune activation seen in autoantibody production and established RA (54). However, it is worth noting that IL-10 was found to upregulate Fc γ receptor expression in monocytes and increase TNF production in response to immune complex stimulation, which may counteract its otherwise antiinflammatory effects and worsen proinflammatory responses in RA (55). Hence, considering our data, the role of IL-10 in the development of RF and progression to RA warrants further study.

Our findings challenge the view that low-titer RF production is necessarily transient or clinically benign (7). Indeed, approximately two-thirds of individuals with low-titer RF positivity (<3× ULN) remained positive after a mean follow-up of 4 years, suggesting that low-titer RF production can be persistent. Furthermore, individuals with low-titer RF positivity had markedly higher rates of RA, indicating that low-titer RF production could have clinical implications. The RF proteomics signature showed titer-dependent associations, including at low titers, without a clear threshold effect. Hence, our data suggest that low-grade RF production is a mild form of immune dysregulation linked with RF mechanisms rather than a transient and benign immunological phenomenon.

Our study has a few limitations. The PheWAS analysis was exploratory in nature and was performed using diagnostic codes from linked medical health records and self-reports, which could have resulted in a misclassification bias. For the UK Biobank cohort, we did not have data on ACPA serostatus, and the RF status was ascertained through immunoturbidimetry, which could not separate between different Ig subtypes of RF. Although we utilized a broad definition of autoimmunity and had clinical data capture across diverse resources such as self-reports and primary care and hospital records, it is possible that some individuals with undiagnosed autoimmune diseases were included in our RF^+^ cohort. GWAS analysis was restricted to individuals from White populations due to the limited sample size available for other populations. Functional studies will be needed to understand which genes and cell types are modulated by the SNPs in genetic loci that showed an association with RF production. While we were able to demonstrate the selective reprogramming of the memory B cell compartment in RF and identify key RF-related markers supported across omics layers, further characterization of the RF-related transcriptional programs will be an important research direction.

In conclusion, our findings reframe RF production as a genetically controlled trait anchored in memory B cell dysregulation with a mucosal inflammation link and uncover the molecular pathways of RF-to-RA progression. Further research will be needed to turn the molecular signatures that we identified into accurate prediction models and to validate the RF- and RA-specific pathways for identification of potential drug targets for RA prevention.

## Methods

### Sex as a biological variable.

Our study examined data from both male and female participants. Biological sex was adjusted in the analytic models as reported below. No sex-specific analysis was performed.

### Study populations and design.

The UK Biobank is a prospective cohort study that enrolled approximately 500,000 individuals aged 40 to 69 years between 2006 and 2010, with extensive molecular profiling and health record linkage (56). Individuals enrolled in the UK Biobank were tested for RF for research purposes at cohort entry as a part of the biomarker panel, with testing being repeated for a subset of individuals in the cohort (~20,000) several years later (56). The ALTRA is a prospective, longitudinal cohort study designed to characterize the immune alterations and molecular pathogenesis occurring during the early, preclinical phase of RA (8). The ALTRA cohort includes individuals at risk for RA, as defined by ACPA positivity, and healthy controls (HCs) without autoimmune disease. ALTRA participants underwent RF testing for research purposes. We performed an original reanalysis of the individual-level scRNA-seq data (8). The details regarding sample processing, assay methodology, and quality controls used for RF testing in the UK Biobank and ALTRA cohorts are described in Supplemental Methods).

### Case selection strategies for RA and autoimmune diseases in the UK Biobank.

We defined asymptomatic RF production in the UK Biobank as individuals who tested positive for RF and did not have any autoimmune disease at cohort enrollment. To maximize sensitivity, we defined the presence of autoimmune disease as any report of autoimmune disease diagnosis in nurse interviews (self-report), hospital admissions, or primary care records (Supplemental List 1). For subgroup analyses involving RF^+^ RA, individuals with RA were defined as having an RA diagnosis code, RF positivity, and self-reported disease-modifying antirheumatic drug (DMARD) use upon inclusion in the cohort (Supplemental List 2). The positive predictive value (PPV) of a similar case selection strategy was estimated as 97% in prior studies (57).

### PheWAS.

We performed a PheWAS using the 3-digit-level International Classification of Diseases (ICD) codes mapped from clinical data obtained during the interview with the nurse at enrollment and linked hospital admission, primary care, and cancer registry records. Details of the data-linking process are described in Supplemental Methods. We defined “prevalent cases” with respect to the RF testing date as those including individuals who had a diagnosis before or within 6 months of sample collection. Individuals who had their first diagnosis at least 6 months after sample collection were considered “incident cases” and were excluded from the analyses. We tested the association between RF status and each prevalent phenotype independently using logistic regression adjusted for age at sample collection, sex, self-reported ethnic background, and smoking status. Individuals with missing covariate data (~0.05% of the whole cohort) and phenotypes with fewer than 30 cases were excluded from the analyses. We controlled the FDR at 10% through the Benjamini-Hochberg (BH) approach (58). These analyses were performed using PheWAS package in R.

### GWAS.

We used genotyping data in the UK Biobank, which includes approximately 850,000 genotyped variants through the UK BiLEVE Axiom array and the UK Biobank Axiom array with TOPMed reference-based imputation. We performed quality control by filtering SNPs with a minor allele frequency (MAF) of less than 1%, significant departures from Hardy-Weinberg (*P* < 1 × 10^–3^), high missingness rates (≥3%), and INFO scores (indicating imputation quality) below 0.9. We also removed individuals with poor genotyping success rates (≤98%), those who were outliers for heterozygosity, had genetic relatedness up to third-degree relatives, were of self-reported mixed ancestral backgrounds, had sex chromosome aneuploidies, or had mismatches between genetic and self-reported sex. Additional details regarding the genetic data in the UK Biobank, imputation processes, and quality controls are described in Supplemental Methods. From the asymptomatic RF^+^ cohort at baseline, we also excluded individuals who received an incident autoimmune disease diagnosis after cohort enrollment. The analysis was restricted to individuals from self-reported White populations, given the limited sample size from other populations. We tested the association between individual SNPs and binary RF status using logistic regression adjusted for age at recruitment, sex, and first 5 principal components (PCs) to control for population stratification. The genome-wide significance was defined as a *P* value of less than 5 × 10^–8^. HLA classical alleles were imputed using the SNP2HLA module of the HLA-TAPAS analysis workflow and Beagle version 5.4 (59). HLA classical alleles with an imputation quality metric R2 of greater than 0.9 were used in subsequent analyses. We performed stepwise logistic regression to test for independent effects of the SNPs and classical HLA alleles in the extended HLA region. The GWAS analysis was conducted using PLINK version 2 software (60).

Lead genome-wide significant variants in each locus were mapped to the closest protein-coding gene followed by protein-protein interaction (PPI) analyses using the STRING (Search Tool for the Retrieval of Interacting Genes/Proteins) database (61). STRING was used to construct a PPI network among input proteins using curated and predicted functional associations (61). To assess whether the observed connectivity exceeded that expected by chance given a random protein set of comparable size, we used STRING’s built-in PPI enrichment test (61). Functional enrichment was then evaluated within STRING for Gene Ontology using the whole genome as background and multiple-testing correction with terms considered significant at an FDR of 0.05 or less with the BH approach (58).

We used the precalculated RA PRS provided by the UK Biobank (62) to compare the RA genetic risk between RF^+^ individuals without RA and RF^–^ HCs. Discrimination performance of the RA PRS was assessed through area under the receiver operating characteristic curve (ROC-AUC) with 95% CIs calculated by the Delong method (63).

### PWAS.

A subset of UK Biobank cohort (~50,000) underwent proteomic profiling through the proximity extension assay (PEA) method of the Olink Explore platform (64). This platform provides the normalized protein expression (NPX) value, which is an arbitrary unit for relative quantification of serum proteins on a log_2_ scale. For quality control, data were preprocessed by the UK Biobank Pharma Proteomics consortium as described in Supplemental Methods. We performed differential protein abundance analyses for each protein by fitting a multivariable linear model with NPX as the dependent variable and binary asymptomatic RF status as the primary independent variable. To further investigate the dose-response relationship between serum proteins and RF levels, a subgroup analysis was performed comparing the following RF^+^ titer groups with RF^–^ individuals: low (<3× ULN), medium (3–12 ULN), and high (>12 ULN). We performed an additional subgroup analysis by excluding individuals who received an incident autoimmune disease diagnosis after cohort enrollment to determine whether the RF proteomics signature was influenced by the proteomic alterations of RA or other autoimmune diseases prior to symptom onset. We also compared RF^+^ individuals without RA to individuals with RF^+^ RA. All models were adjusted for age, sex, ethnic background, processing time, and batch to account for demographic and technical variations. Missing data for each protein were imputed with the respective median across all samples. The analysis utilized linear models with empirical Bayes moderation of standard errors (65). Differential expression statistics were obtained in the form of log fold changes (logFCs) with their associated moderated *t* statistics and *P* values for each protein. Proteins with an absolute logFC of at least 0.05 and FDR <1% through the BH approach were considered significant. All analyses were conducted in R (version 4.4.2) using the limma package (version 3.60.6) (65). Gene set overrepresentation analysis was performed for proteins with a differential abundance via Reactome (version 7.2) using WebGestaltR (version 0.4.6, method = ORA), with all measured proteins by the Olink Explore panel being used as background genes and the FDR controlled at 5% by the BH approach (58, 66, 67).

### Pseudobulk differential gene expression analysis.

We used the preprocessed ALTRA cohort scRNA-seq data, which underwent extensive quality control. The quality controls applied for the ALTRA cohort are described in Supplemental Methods. Using the first collected sample for each participant, we performed pseudobulk differential expression analysis for each immune cell type, comparing RF^+^ with RF^–^ individuals without RA at the time of sample collection, adjusting for age at sample collection, sex, BMI, ACPA status, and batch B182 status (as recommended by the authors of the original analysis, given the significant batch effects observed with this particular batch) (8). Immune cell type labels were assigned using Celltypist model with the Allen Institute for Immunology (AIFI) Immune Cell Atlas used as a reference (17). We also performed the same analysis comparing ACPA^+^ and ACPA^–^ individuals without RA, adjusted for RF status using the same model structure. The FDR was controlled at 10% through the Storey-Tibshirani procedure. All differential expression analyses were performed using the PyDESeq2 package (68, 69).

### Immune cell type population differential abundance analysis.

We utilized the precalculated centered log ratios (CLRs) for immune cell type populations from the ALTRA cohort. The CLR was calculated by extracting counts and frequencies for each cell subset from the scRNA-seq data followed by the addition of a pseudocount of 1 to raw counts and CLR transformation. We performed linear regressions to compare the CLR values for each immune cell type across RF^+^ and RF^–^ individuals without RA, adjusting for age at sample collection, sex, BMI, and ACPA status. The FDR was controlled at 10% through the BH approach. Further details regarding data processing in the ALTRA cohort have been published elsewhere (8).

### Gene set enrichment analysis of cytokine response signatures.

We performed a signed preranked gene set enrichment analysis (GSEA) utilizing the Human Cytokine Atlas dataset, which provides data on cytokine-induced transcriptomic signatures for 90 different cytokines in immune cells (70). For each mapped cell type and cytokine, cytokine-responsive genes were defined as those with an adjusted *P* value of less than 0.01 and an absolute logFC of greater than 0.8 and were further partitioned into cytokine-upregulated and cytokine-downregulated subsets. To encode directionality within a single bidirectional gene set, the ranked query statistic was transformed on a per-cytokine basis, such that genes upregulated by the cytokine retained their original DESeq2 statistic, whereas genes downregulated by the cytokine were multiplied by –1, and all other genes were left unchanged and served as the background. For each query cell type, genes were ranked using the DESeq2 test statistic from the pseudobulk differential expression analysis. Cytokine signatures containing fewer than 10 total responsive genes were excluded. The union of upregulated and downregulated cytokine-responsive genes was tested as a single gene set using preranked GSEA with 1,000 permutations (71). A normalized enrichment score (NES) indicated concordant enrichment of the cytokine signature, with cytokine-induced genes enriched among pseudobulk upregulated genes and cytokine-repressed genes enriched among pseudobulk downregulated genes. Only cytokines with statistically significant enrichment in at least 2 different cell types with concordant effect directions were considered.

### Study approval.

This research was conducted using the UK Biobank resource under application no. 145447. The UK Biobank has received ethics approval from the North West Multi-Centre Research Ethics Committee (REC) (REC reference: 16/NW/0274). All study participants provided informed consent at the time of enrollment.

### Data availability.

Data derived from the UK Biobank, including raw sequencing data, are available upon application through the UK Biobank. The ALTRA cohort data are publicly available at https://apps.allenimmunology.org/aifi/insights/ra-progression/downloads/ Raw sequencing data from the ALTRA cohort are available at dbGaP (phs003944.v1.p1).

## Author contributions

MH was responsible for conceptualization, funding acquisition, investigation, methodology, and writing of the original draft of the manuscript. AHS was responsible for conceptualization, methodology, supervision, and review and editing of the manuscript.

## Conflict of interest

The authors have declared that no conflict of interest exists.

## Funding support

Arthritis Foundation through the 2025 Rheumatoid Arthritis Research Program Pilot Award (to MH).

## Role of funding source

The funding source had no role in the study design; in the collection, analysis and interpretation of data; in the writing of the report; or in the decision to submit the article for publication.

## Supplementary Material

Supplemental data

ICMJE disclosure forms

Supplemental table 1

Supplemental table 3

Supplemental table 4

Supplemental table 5

Supplemental table 6

Supplemental table 7

Supplemental table 9

Supplemental table 10

Supplemental table 11

## Figures and Tables

**Figure 1:**
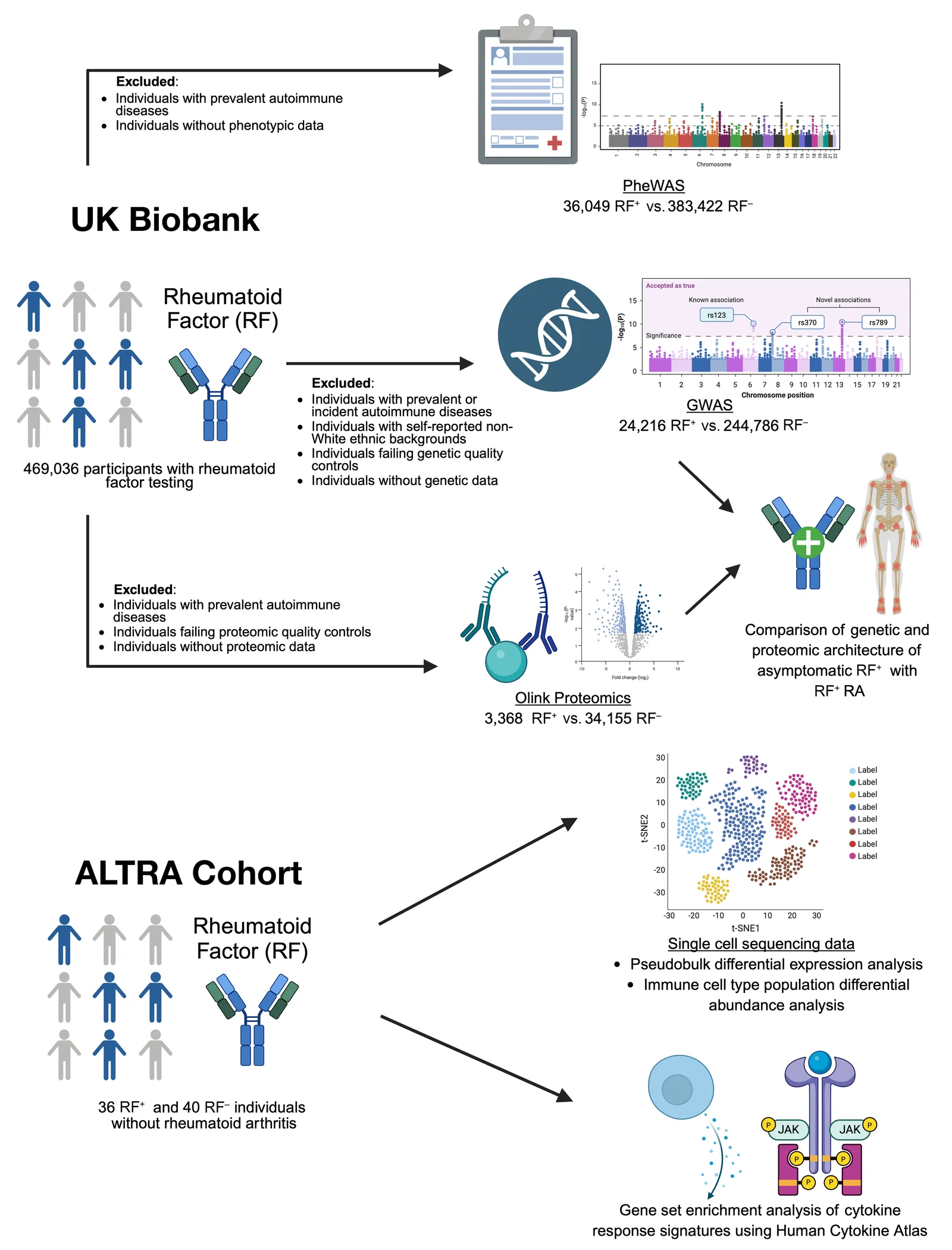
Diagram of the study design.

**Figure 2:**
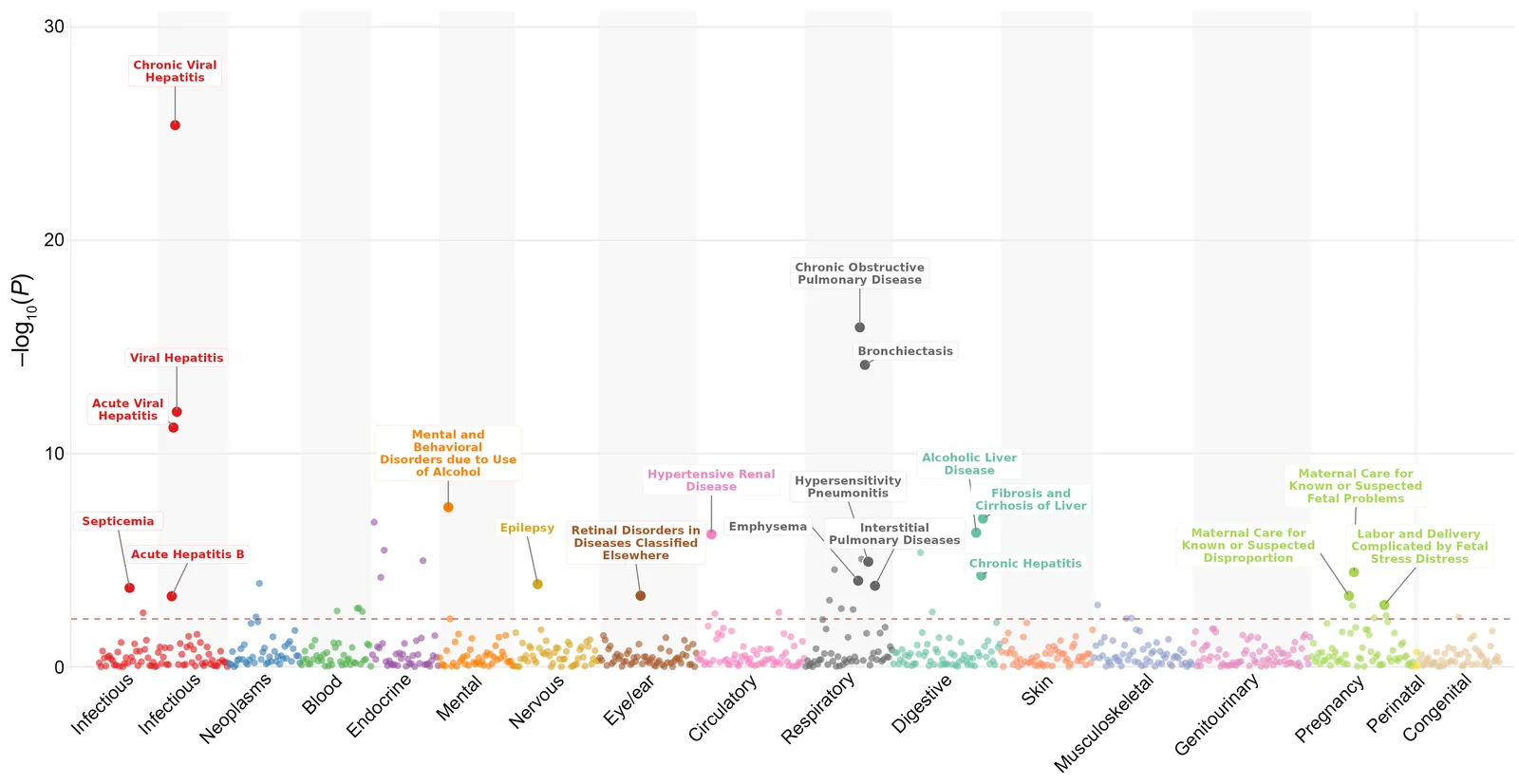
Manhattan plot for PheWAS of RF positivity among individuals without an autoimmune disease diagnosis in the UK Biobank. The *x* axis displays individual phenotypes grouped by clinical category, and the *y* axis shows –log_10_ *P* values. Each semitransparent point represents a distinct phenotypic trait, color-coded by its respective clinical domain. Alternating background shading delineates these broader categories. The horizontal dashed red line indicates the BH-corrected FDR of a 10% significance threshold. To emphasize the most robust findings, the top-20 most significant associations that also have a clinically notable effect size (OR >1.2 or <0.83) are highlighted with larger, outlined markers and explicitly labeled with their condition descriptions.

**Figure 3:**
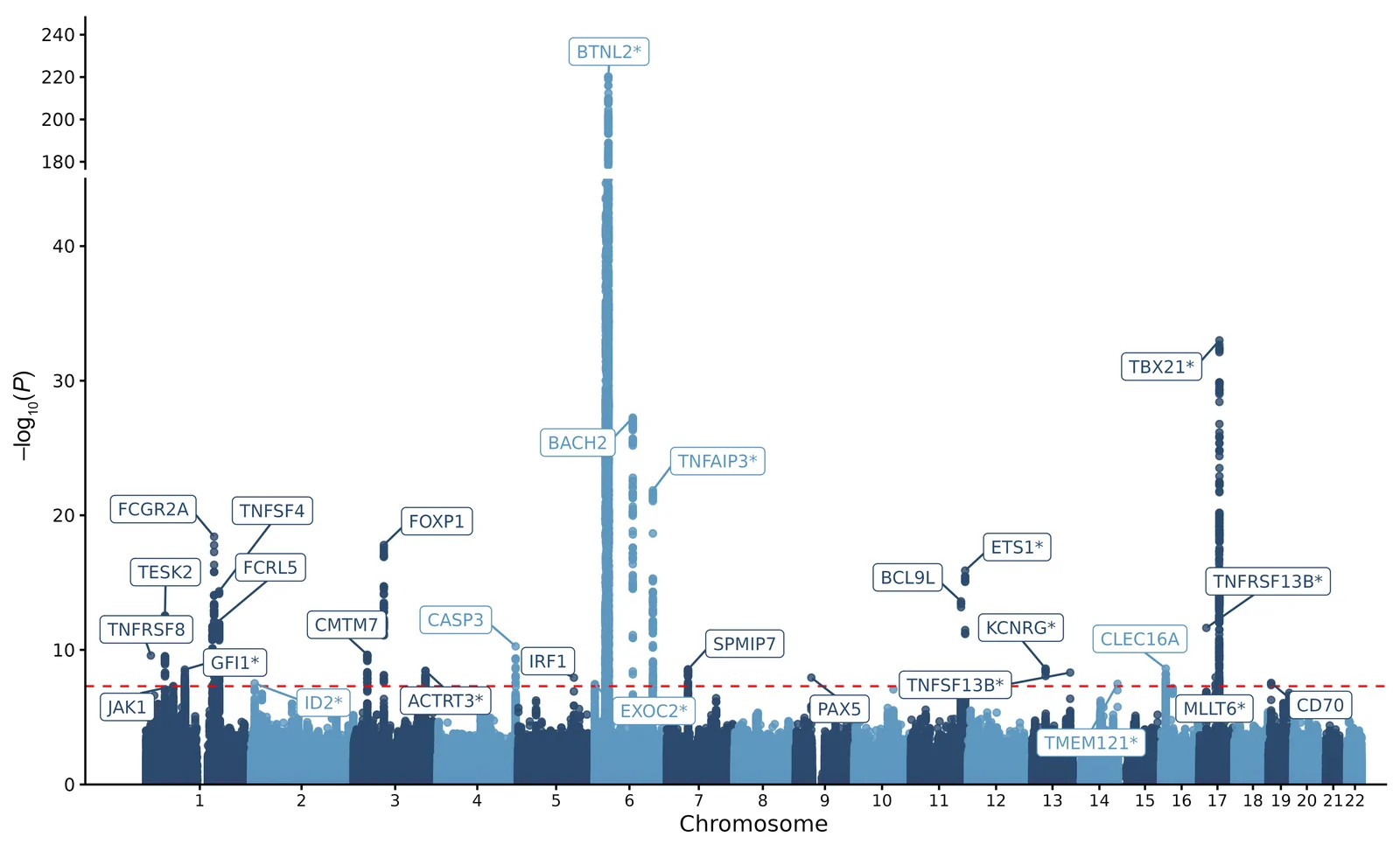
Manhattan plot for a GWAS of RF positivity among individuals without an autoimmune disease diagnosis in the UK Biobank. The *y* and *x* axes refer to the –log_10_ *P* values and chromosome positions, respectively. The red horizontal line represents the genome-wide association threshold (*P* < 5 × 10^−8^). Blue and dark blue dots represent individual genetic variants. Variants with a significant association at the genome-wide level were annotated to the closest protein-coding gene based on the hg38 build. Asterisks label loci with intergenic variants mapped to the closest protein-coding gene, whereas the remaining variants are located within the annotated gene.

**Figure 4:**
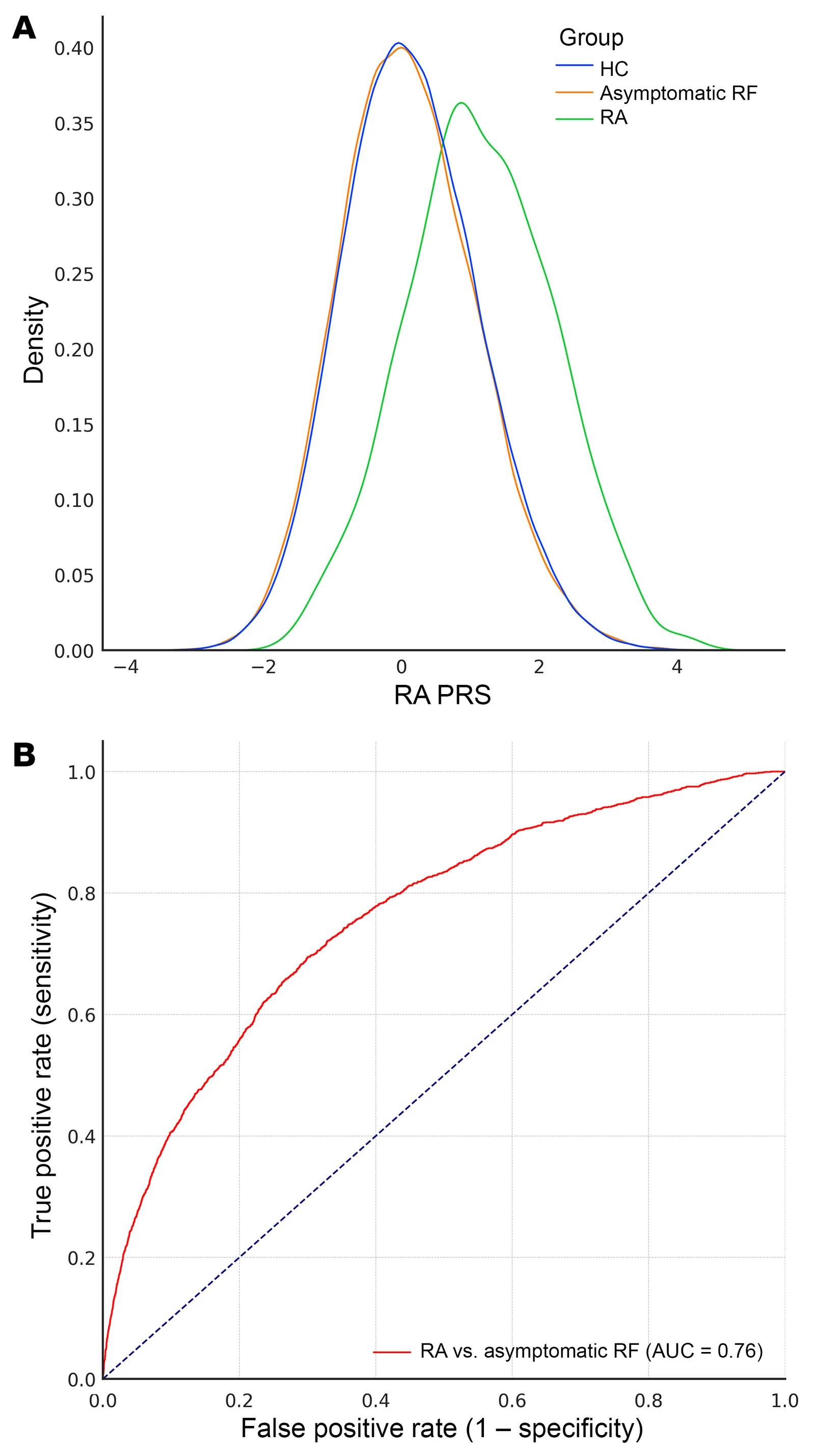
Distribution and discriminative performance of the RA PRS among individuals with asymptomatic RF production. (**A**) Density plot illustrating the distribution of the RA PRS across the study groups. (**B**) ROC curve evaluating the ability of the RA PRS to discriminate between patients with RF^+^ RA and asymptomatic RF^+^ individuals. The red solid line indicates the model’s performance, with the AUC reported in the legend. The dashed navy line represents the baseline of a random classifier (AUC = 0.50).

**Figure 5:**
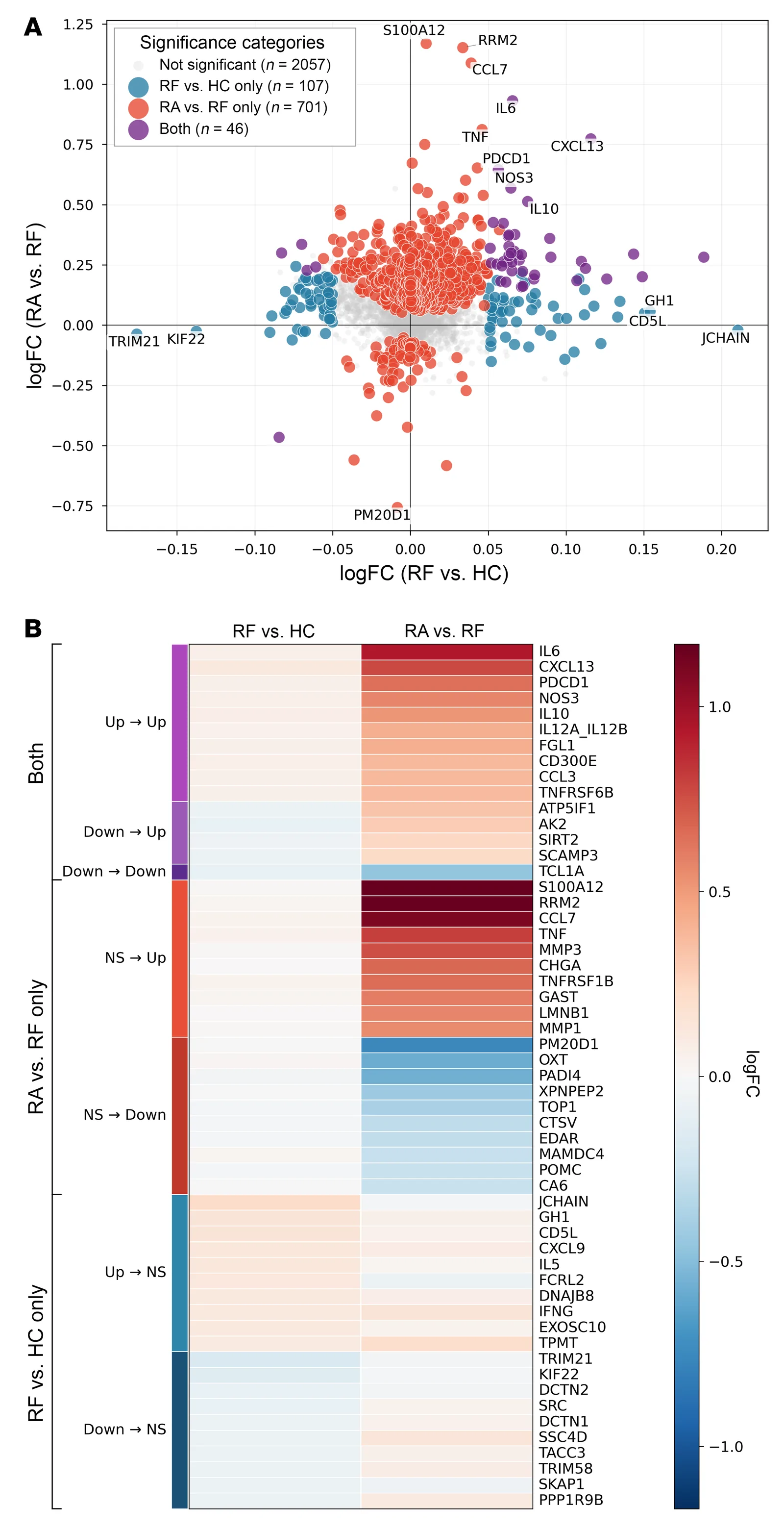
Comparative proteomics profiling of asymptomatic RF production and RF^+^ RA. (**A**) Scatter plot of protein-level logFCs in asymptomatic RF^+^ versus RF^–^ HCs (*x* axis) versus RF^+^ RA versus RF^+^ without RA (*y* axis). Proteins were classified using a combined significance criterion requiring both an adjusted *P* value of less than 0.01 and |logFC| of greater than 0.05 in each comparison. Points are colored according to the significance category: RF versus HCs only (significant only in RF vs. HCs), RA versus RF only (significant only in RA vs. RF), both (significant in both comparisons), and not significant. Proteins that were top hits defined by absolute logFC within single-comparison groups and by Euclidean distance for proteins significant in both comparisons are annotated in the plot. (**B**) Heatmap of proteins showing logFC values for RF versus HCs and RA versus RF comparisons. Proteins are grouped by the direction of change across comparisons and are ranked within direction groups by their logFC magnitude. Up to 10 proteins with the most statistically significant change in each direction group are depicted. “Up” and “Down” represent upregulation and downregulation in the respective analyses, while “NS” represents no statistically significant change. Direction group names such as “Up → Up” or “Up → Down” represent the direction of change for the depicted protein in RF versus HCs and RA versus RF comparisons, respectively. Cell colors represent the logFC magnitude and direction (centered at 0).

**Figure 6:**
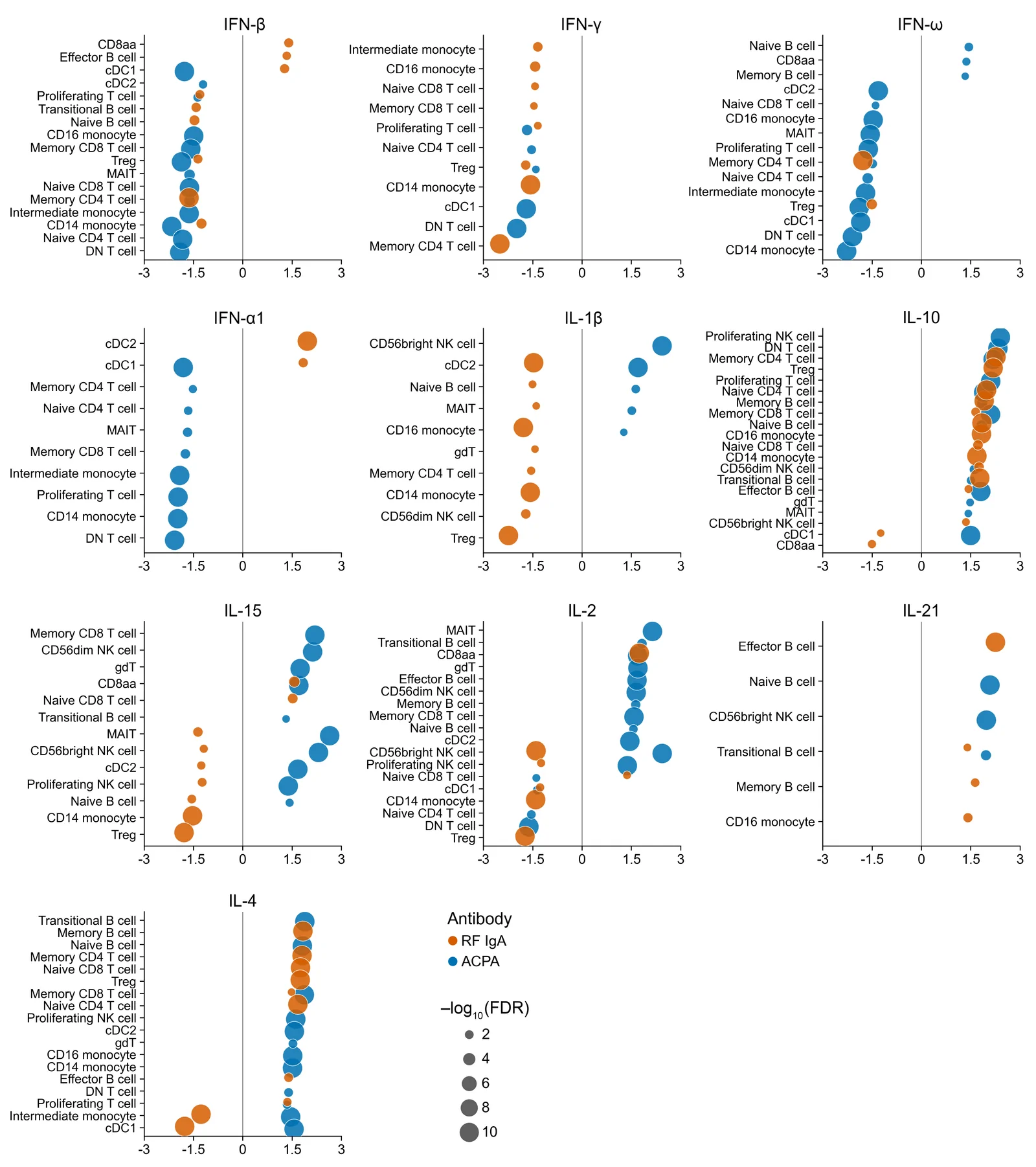
Cell-type–specific enrichment of cytokine signatures associated with IgA RF and ACPAs. Dot plot displaying the NES for targeted cytokine gene signatures across immune cell populations. GSEA was performed independently for IgA RF and ACPA pseudobulk differential expression analysis results. The *x* axis represents the NES, where positive and negative values indicate up- and downregulation of the respective cytokine signatures. Dot color denotes the associated autoantibody (orange for IgA RF, blue for ACPA). The size of each dot is proportional to the statistical significance of the enrichment.

**Table 1:**
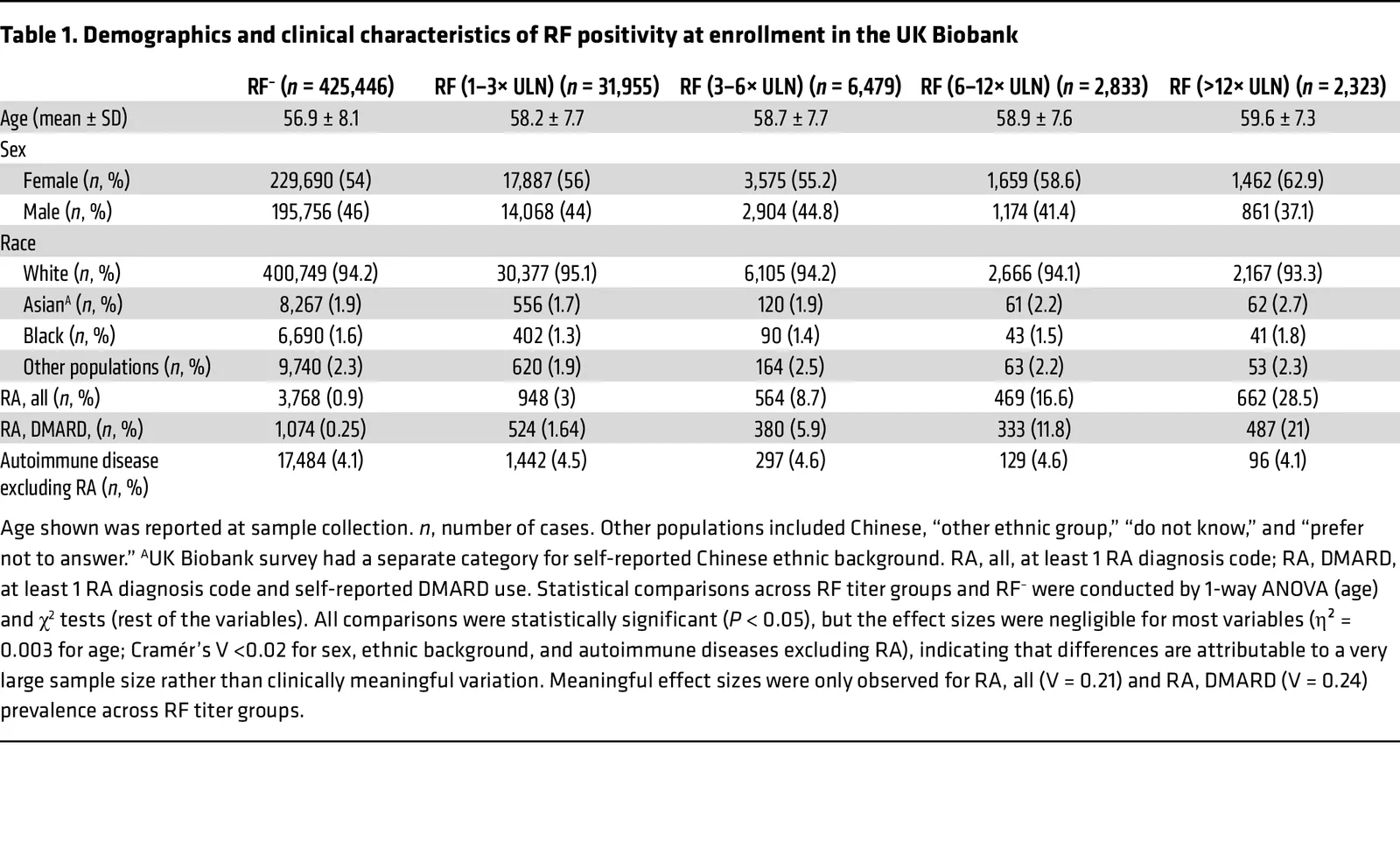
Demographics and clinical characteristics of RF positivity at enrollment in the UK Biobank

## Version 1 06/30/2026

In-Press Preview

## Version 2 10/01/2026

Electronic publication
