# Supplementary material for: Rheumatoid factor production is genetically and molecularly distinct from rheumatoid arthritis
Source: J Clin Invest. 2026 Jun 30;136(19):e208390. doi: 10.1172/JCI208390 (PMC13626823; doi:10.1172/JCI208390)
Supplement: Supplementary file 1 [file jci-136-208390-s174.pdf]

SUPPLEMENTAL MATERIAL

Supplemental Figure 1. Sankey diagram illustrating rheumatoid factor status transitions at follow-up testing.

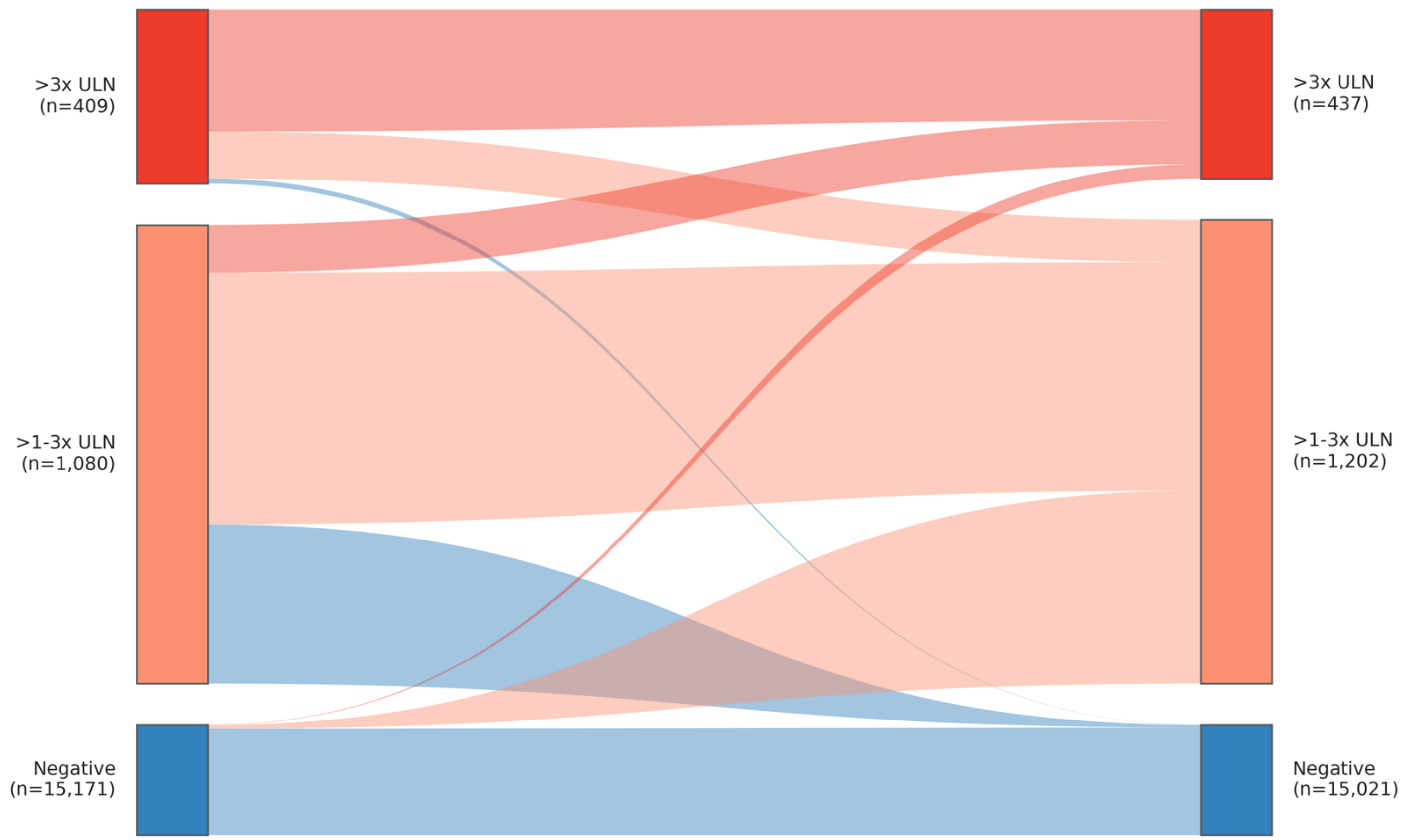

Individuals in the UK Biobank who underwent repeat RF testing (N=16,660) categorized by their baseline RF titer group (left) and their follow-up RF titer group at repeat testing (right). The thickness of the connecting bands represents the absolute number of patients transitioning between states. Positive nodes are magnified in the graph for both baseline and repeat testing to help with visual interpretation while keeping the proportionality among the positive group nodes. The absolute number of individuals in each node was provided in the graph. RF, rheumatoid factor; ULN, upper limit of normal.

Supplemental Figure 2. Locus plot depicting the association of genetic variants within the extended HLA region with rheumatoid factor production.

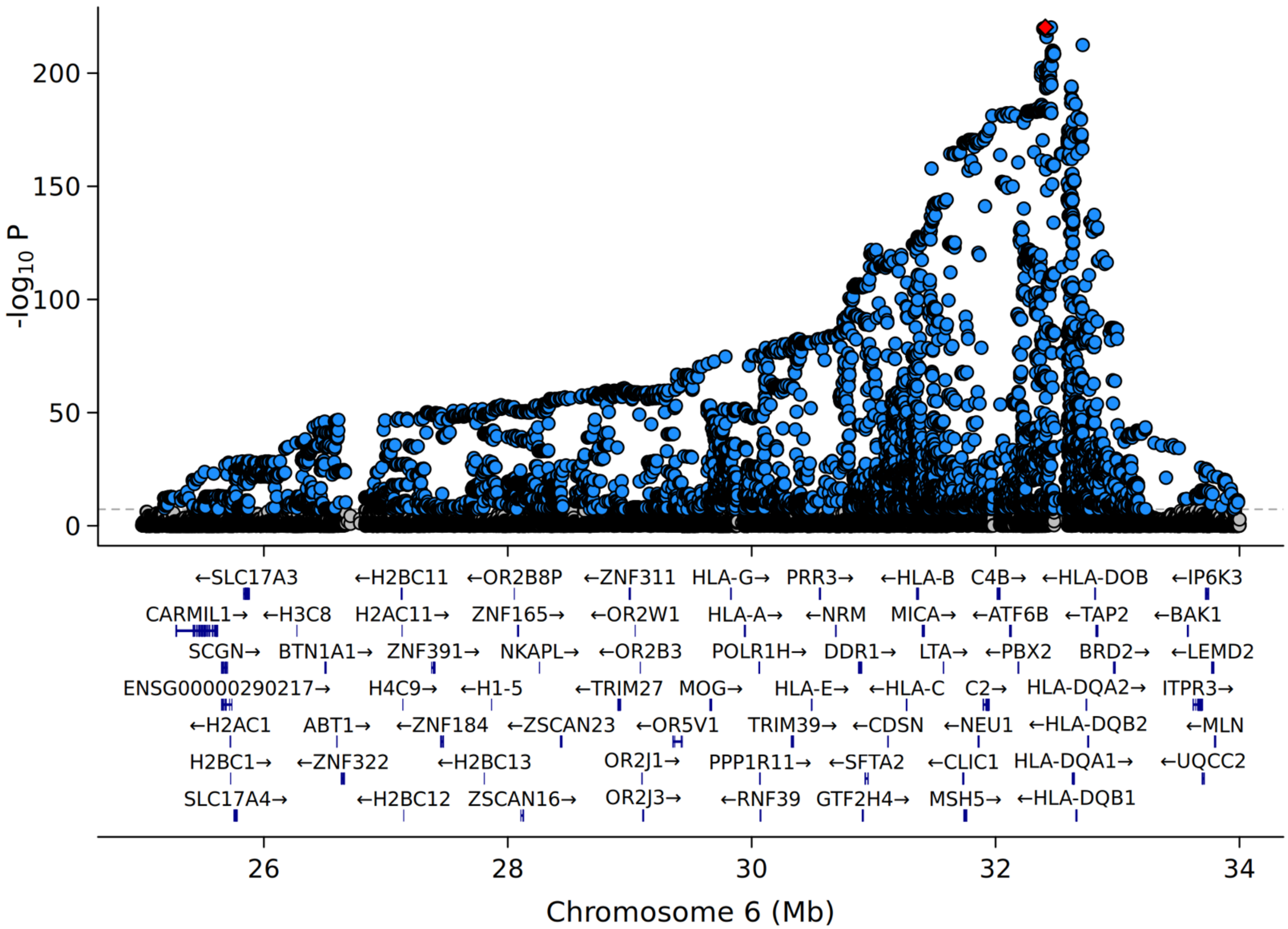

log10 *P*-values (y-axis) against their physical genomic position in megabases (Mb) on chromosome 6 (x-axis). Each circle represents a single genetic variant. The red diamond highlights the lead variant exhibiting the strongest statistical association in this locus. The horizontal dashed gray line indicates the threshold for genome-wide significance. The legend illustrates the annotated genes located within this chromosomal window, with arrows denoting the direction of transcription.

Supplemental Figure 3. Protein-protein interaction network and functional enrichment analysis of rheumatoid factor associated genetic risk loci.

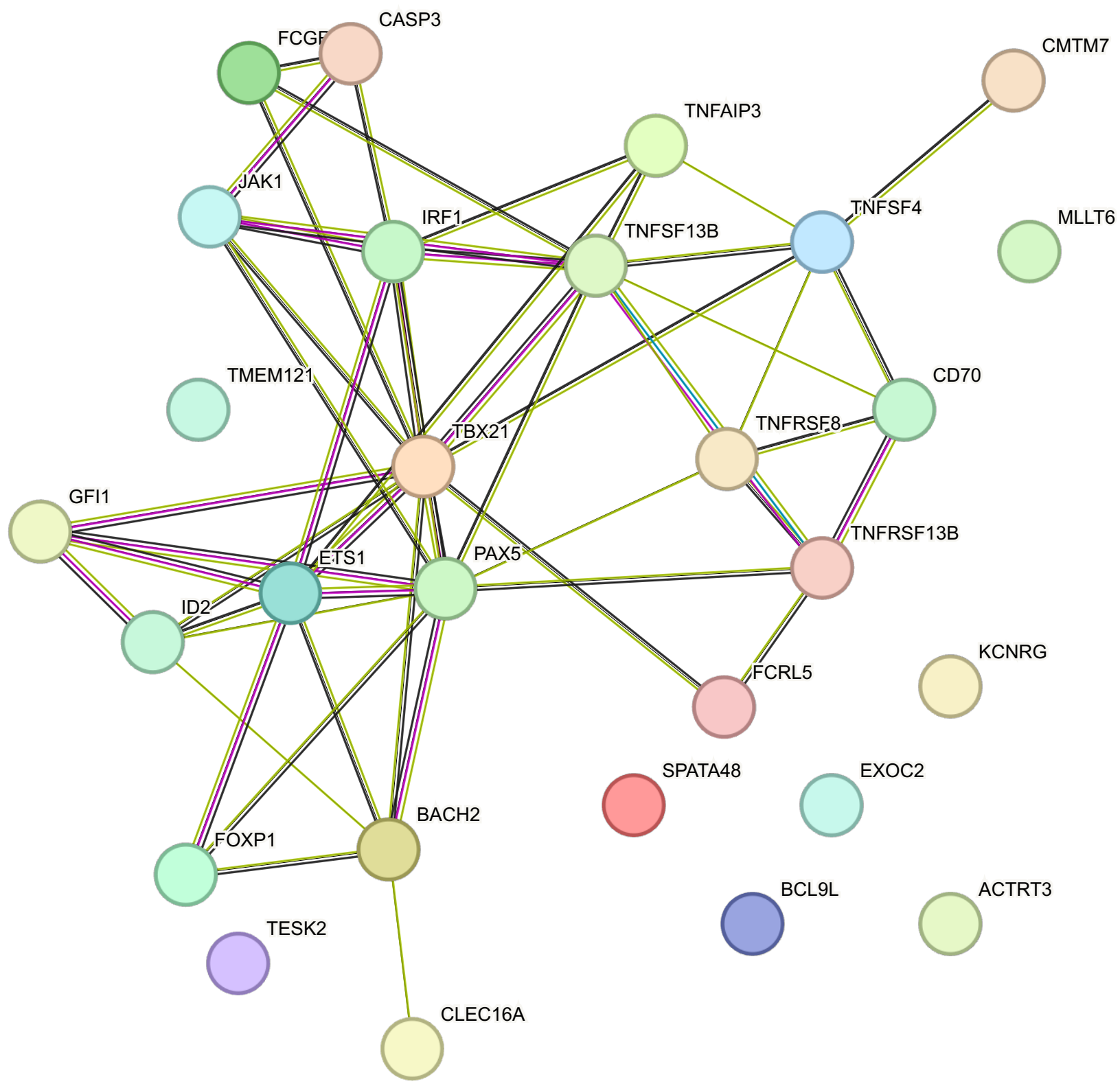

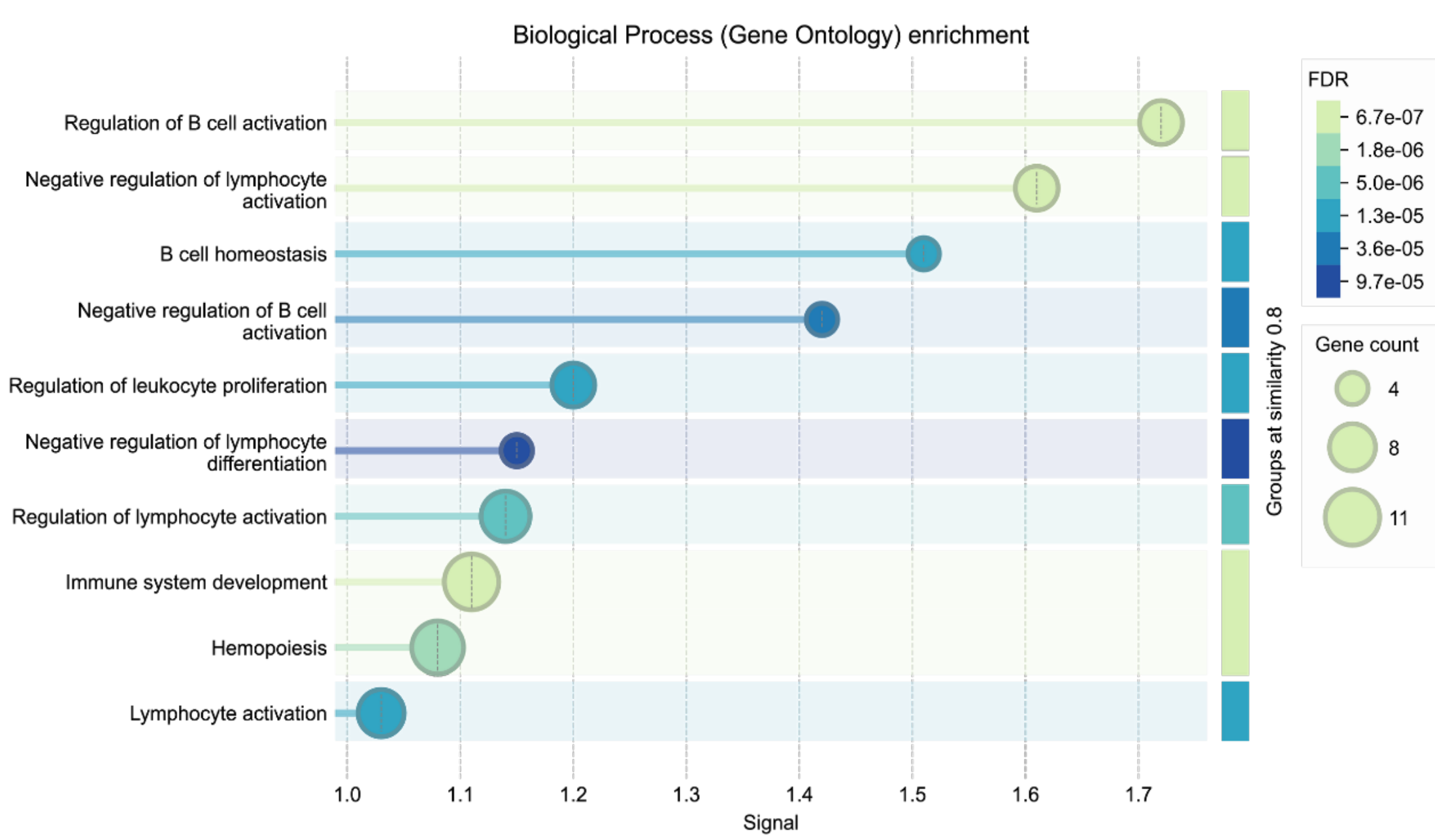

Upper panel, protein-protein interaction (PPI) network constructed using the STRING database illustrating the functional and physical associations between the encoded proteins of genetic risk loci. Individual nodes represent proteins, while the connecting edges denote interactions. Lower panel, functional enrichment analysis of the RF genetic risk loci. The plot displays the top enriched gene ontology (GO) terms on the y-axis. The size of each circle corresponds to the number of genes from the input list associated with that specific term, with larger circles indicating a higher number of involved genes. The color gradient of the circles and bars represents the false discovery rate (FDR) for the enrichment. For *SPMIP7*, we used the alternate gene alias *SPATA48* as this was the name of the corresponding gene entry in the STRING database.

Supplemental Figure 4. Density plot depicting the distribution of the rheumatoid arthritis polygenic risk score (RA-PRS) among individuals with asymptomatic rheumatoid factor (RF) production stratified based on RF titers.

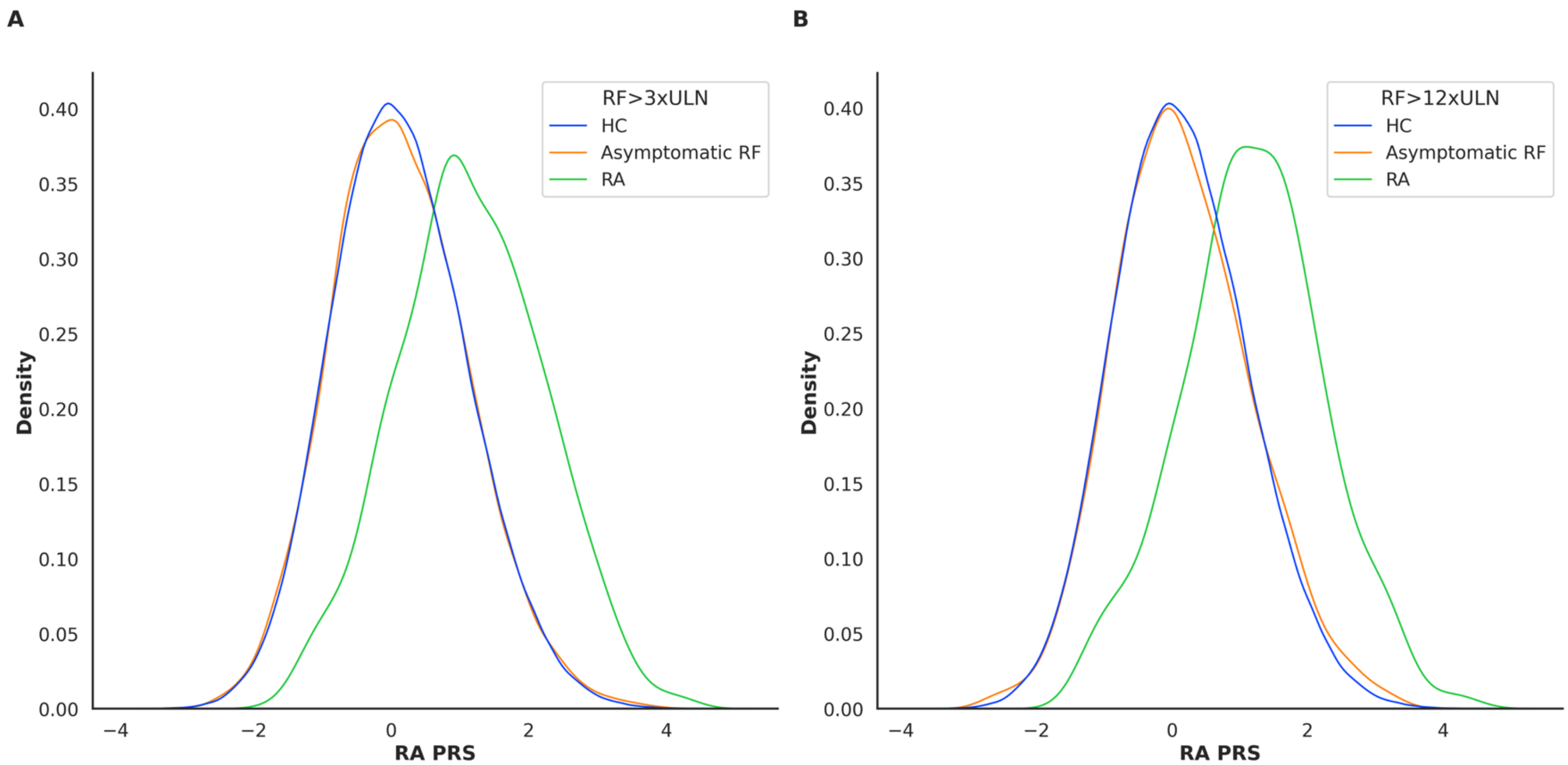

ULN, upper limit of normal. RF, rheumatoid factor. RA, rheumatoid arthritis. PRS, polygenic risk score.

Supplemental Figure 5. Enrichment analysis results of proteomic changes associated with rheumatoid factor (RF)-positive versus RF-negative healthy individuals, and RF-positive RA versus asymptomatic RF-positive individuals.

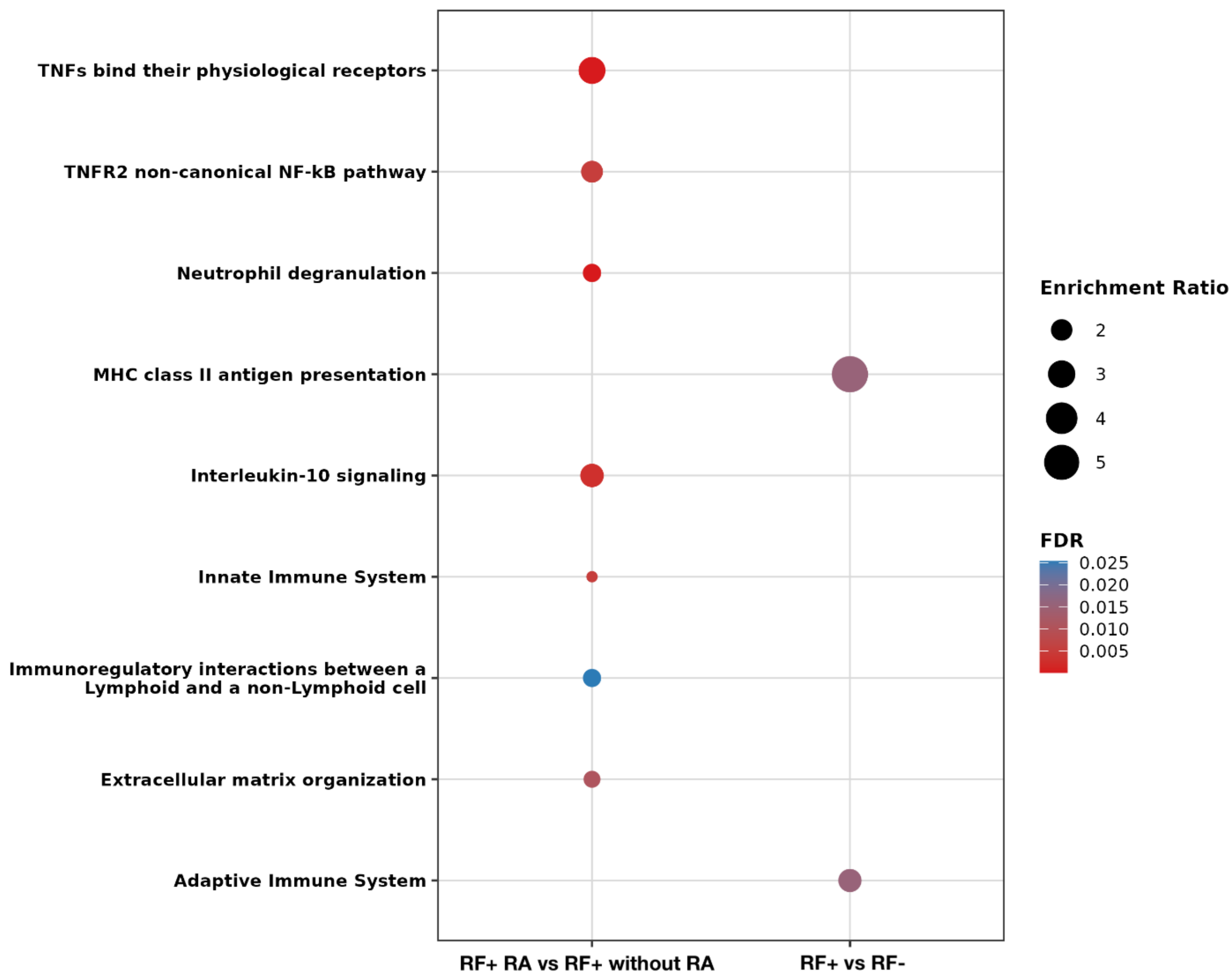

The dot plot visualizes the significantly enriched biological pathways derived from differential protein abundance profiles. The y-axis depicts the enriched Reactome pathway terms. The size of each node is proportional to the enrichment ratio, indicating the magnitude of pathway enrichment. The node color represents the statistical significance based on the false discovery rate (FDR), with red indicating a lower FDR and blue representing a higher FDR.

Supplemental Figure 6. Pseudobulk differential expression analysis results across immune cell types in ACPA-positive and RF-positive individuals.

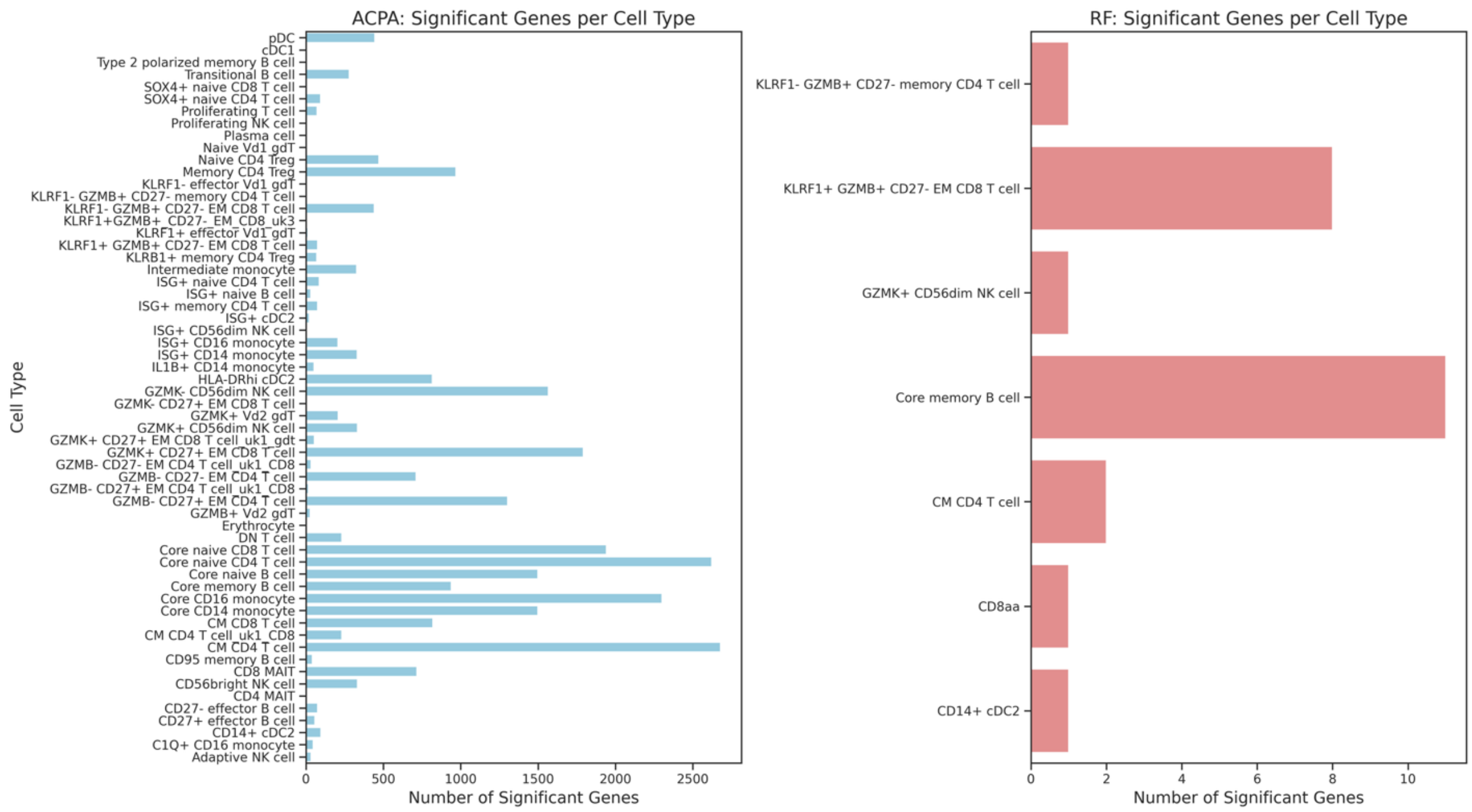

Bar plots representing the total number of significantly differentially expressed genes (DEGs), at false discovery rate of 0.1, identified via pseudobulk single cell RNA-sequencing analysis across immune cell populations. (A) The left panel illustrates the count of significant DEGs associated with anti-citrullinated protein antibodies (ACPA) status. (B) The right panel displays the count of significant DEGs associated with rheumatoid factor (RF) status. Note that the x-axes are scaled independently to account for the substantially higher transcriptional yield in the ACPA comparison and to ensure the visibility of the RF-associated DEGs.

Supplemental Table 2. Pairwise conditional analysis results between rs3129959 and HLA-DQA1:05 in rheumatoid factor (RF)-positive compared to RF-negative individuals among those without clinical autoimmune disease diagnosis.

Marker	Covariates	*P*-value	OR (95% CI)
rs3129959	None	$5.4x10^{-221}$	1.45 (1.42-1.48)
	HLA-DQA1:05	$1.5x10^{-98}$	1.35 (1.31-1.39)
HLA-DQA1:05	None	$2.3x10^{-135}$	1.3 (1.28-1.33)
	rs3129959	$1.4x10^{-18}$	1.12 (1.09-1.15)

CI, confidence interval; OR, odds ratio.

Supplemental Table 8. Demographics of ALTRA cohort participants with RF testing and available single cell sequencing data included in the analysis.

	RF-negative (n = 40)	RF-positive (n = 36)
Age at sample collection (years)	59.68 (15.97)	53.11 (16.01)
BMI (kg/m²)	28.07 (5.57)	26.18 (4.59)
**Biological sex**		
Female	31 (77.5%)	31 (86.1%)
Male	9 (22.5%)	5 (13.9%)
**RF IgM**		
Negative	40 (100.0%)	4 (11.1%)
Positive	0 (0.0%)	32 (88.9%)
**RF IgA**		
Negative	40 (100.0%)	20 (55.6%)
Positive	0 (0.0%)	16 (44.4%)

RF, rheumatoid factor; BMI, body mass index.

Supplemental Methods

Rheumatoid factor testing in UK Biobank and ALTRA cohorts

All rheumatoid factor (RF) testing in the UK Biobank was performed by the Beckman Coulter AU5800 analytical platform which uses the immuno-turbidimetric method and reports RF levels between the limits of detection of 10 and 120 IU/ml. Accordingly, a 10 IU/ml threshold was used as the overall positivity threshold. Further details regarding assay methodology and biomarker data processing in the UK Biobank are described in the following resources: 1) https://biobank.ndph.ox.ac.uk/ukb/refer.cgi?id=1227 and 2) https://biobank.ndph.ox.ac.uk/ukb/refer.cgi?id=5636.

RF testing in the ALTRA cohort was performed for IgM and IgA subtypes separately using an ELISA platform (QuantaLite, Werfen) with cut-offs for positivity based on the clinical laboratory's internal cut-offs (>1.7 International Units for RFIgA and >6.7 International Units for RFIgM). Accordingly, we defined RF positivity based on these thresholds as having either a positive result in RF-IgA or RF-IgM. Further details regarding cohort recruitment and data collection in ALTRA have been published elsewhere (1).

Clinical data linking process in UK Biobank

The clinical data in the UK Biobank are captured through three different resources: 1) self-report during nurse interviews, 2) hospital admission and death records and 3) primary care records (available for ~45% of the cohort). The conditions reported through these sources are then mapped to 3-digit level ICD-10 codes for each condition excluding cancer outcomes. The overall details regarding the mapping process including the maps for each 3-digit level condition are described in the following resource:

1) General description:
   https://biobank.ndph.ox.ac.uk/showcase/label.cgi?id=1712

2) Mapping for the self-report data:
   https://biobank.ndph.ox.ac.uk/showcase/coding.cgi?id=609
3) Mapping for the primary care data:
   https://biobank.ndph.ox.ac.uk/showcase/coding.cgi?id=1834
   https://biobank.ndph.ox.ac.uk/showcase/coding.cgi?id=1835
4) Mapping between hospital ICD-9 and ICD-10 based codes
   https://biobank.ndph.ox.ac.uk/showcase/coding.cgi?id=1836

As cancer was excluded from this mapping, cancer status for the participants was instead identified through the linked cancer registry data in the UK Biobank. The details of the cancer registry data linkage are available in the following resource: https://biobank.ndph.ox.ac.uk/showcase/label.cgi?id=100092. Subsequently, each individual level ICD-based diagnosis was collapsed to its respective 3-digit level group ICD code for the cancer outcomes similar to the approach described above for non-cancer outcomes.

Imputation and quality controls for GWAS

The details of the genetic data available in the UK Biobank are described in the following resource: https://biobank.ndph.ox.ac.uk/showcase/label.cgi?id=263. We utilized the previously imputed data based on the TOPmed reference panel. Imputation was performed by the TOPMed Informatics Research Center by phasing the UK Biobank genetic data (carried out on 81 chromosomal chunks using Eagle v.2.4). Imputation was performed using Minimac4 v1.0.2 (https://genome.sph.umich.edu/wiki/Minimac4). Imputation was performed in 1Mb chunks and merged back together by chromosome. Further details regarding the imputation process can be found at https://biobank.ndph.ox.ac.uk/showcase/field.cgi?id=21007 and https://biobank.ndph.ox.ac.uk/showcase/label.cgi?id=100319. The description of the underlying genetic data is available in the following paper (2). After quality controls as described in the main methods, we hard-called imputed genotypes by setting the imputed allele probabilities

within 0.1 of its nearest integer with the rest set to missing. The genetic relatedness up to 3rd degree relatives corresponding to a kinship score of 0.0442 was identified using pre-calculated kinship scores provided by the UK Biobank (2, 3). The details for identifying individuals who are outliers for heterozygosity, are of self-reported mixed ancestral backgrounds, have sex chromosome aneuploidies or have mismatches between genetic and self-reported sex have been published elsewhere (2).

Quality controls for proteomic data

The UK Biobank proteomic data underwent quality controls by removing the outlier samples, defined as being more than 5 standard deviations away in any of the following: principal component analysis, sample median and interquartile range. Samples with likely sex swaps indicative of manual handling errors and those with assay warnings by the manufacturer were also removed. As an additional quality control, we removed samples and proteins with more than 20% missing data from our analysis. Further details regarding data collection and quality control processes by the UK Biobank are described in the following resource: https://biobank.ndph.ox.ac.uk/showcase/label.cgi?id=1839. The description of the underlying proteomic data is available in the following paper (4).

Quality controls for single-cell RNA sequencing

High-quality cells were selected by the following criteria: <10% mitochondrial reads, number of genes detected between 200 and 5,000, RNA unique molecular identifiers (UMIs)/cells between 1,500 and 750,000 (1). Doublets were removed using Scrublet with default parameters followed by manual review and removal of Leiden clusters with gene expression profiles indicative of doublets. As done in the original analysis (1), we removed sample-cell type pairs with fewer than 10 cells or 1,000 total genes, genes expressed in fewer than 10% of cells or in less than one-third of samples or with fewer than 15 counts across all samples. Pseudobulking was performed

for L2 and L3 hierarchical levels of immune cell types from the AIFI Immune Cell Atlas. Immune cell type labels were assigned using the Celltypist model with AIFI Immune Cell Atlas used as reference. Manual review of clusters with distinct gene profiles within each predicted cell type was performed and unique labels were assigned to differentiate them from the predicted cell types. Further details regarding quality controls and experimental procedures have been published elsewhere (1).

Supplemental List 1. List of codes used to define autoimmune diseases to be excluded from the study.

1. Rheumatoid arthritis (M05 & M06)
2. Systemic lupus erythematosus (M32)
3. Inflammatory bowel diseases (K50 & K51)
4. Multiple sclerosis (G35)
5. Systemic sclerosis (M34)
6. Sarcoidosis (D86)
7. Dermatopolymyositis (M33)
8. Polyarteritis nodosa and related conditions (M30)
9. Ankylosing spondylitis (M45)
10. Other systemic involvement of connective tissue (M35)
11. Type I Diabetes Mellitus (E10)
12. Other inflammatory spondylopathies (M46)
13. Other necrotizing vasculopathies (M31)
14. Other disorders of the adrenal gland (E27)
15. Psoriatic and enteropathic arthropathies (M07)
16. Acquired hemolytic anemia (D59)
17. Other inflammatory liver diseases (K75)
18. Thyroiditis (E06)
19. Lupus erythematosus (L93)

Supplemental List 2. List of medications used to define the disease-modifying antirheumatic drugs (DMARD) or corticosteroid use for RA definition.

1. Hydroxychloroquine
2. Methotrexate
3. Prednisone
4. Azathioprine
5. Leflunomide
6. Sulfasalazine
7. Adalimumab*

*UK Biobank intake survey did not collect data on other biologics, so only adalimumab was included.

References:

1. He Z, Glass MC, Venkatesan P, Feser ML, Lazaro L, Okada LY, et al. Progression to rheumatoid arthritis in at-risk individuals is defined by systemic inflammation and by T and B cell dysregulation. Sci Transl Med. 2025;17(817):eadt7214.
2. Bycroft C, Freeman C, Petkova D, Band G, Elliott LT, Sharp K, et al. The UK Biobank resource with deep phenotyping and genomic data. Nature. 2018;562(7726):203-9.
3. Manichaikul A, Mychaleckyj JC, Rich SS, Daly K, Sale M, Chen WM. Robust relationship inference in genome-wide association studies. Bioinformatics. 2010;26(22):2867-73.
4. Sun BB, Chiou J, Traylor M, Benner C, Hsu YH, Richardson TG, et al. Plasma proteomic associations with genetics and health in the UK Biobank. Nature. 2023;622(7982):329-38.
